# Impact of Alternative Maternal Demand-Side Financial Support Programs in India on the Caesarean Section Rates: Indications of Supplier-Induced Demand

**DOI:** 10.1007/s10995-015-1810-2

**Published:** 2015-08-11

**Authors:** Lennart Bogg, Vishal Diwan, Kranti S. Vora, Ayesha DeCosta

**Affiliations:** Department of Public Health Sciences, Karolinska Institutet, Stockholm, Sweden; School of Health, Care and Social Welfare, Malardalen University, Vasteras, Sweden; Department of Public Health and Environment, R.D. Gardi Medical College, Ujjain, M.P. India; Indian Institute of Public Health, Gandhinagar, Gujarat India

**Keywords:** Maternal and child health, Caesarean, Institutional delivery, Health insurance, India

## Abstract

**Background:**

This paper examines two state-led public–private demand-side financial support programs aiming to raise hospital delivery rates in two neighbouring Indian states—Gujarat and Madhya Pradesh. The national Janani Suraksha Yojana (JSY) was complemented with a public–private partnership program Janani Sahayogi Yojana (JSaY) in Madhya Pradesh in which private obstetricians were paid to deliver poor women. A higher amount was paid for caesarean sections (CS) than for vaginal deliveries (VD). In Gujarat state, the state program Chiranjeevi Yojana (CY) paid private obstetricians a fixed amount for a block 100 deliveries irrespective of delivery mode. The two systems thus offered an opportunity to observe the influence of supplier-induced demand (SID) from opposite incentives related to delivery mode.

**Methods:**

The data from the two programs was sourced from the Departments of Health and Family Welfare, Governments of Gujarat and Madhya Pradesh, India.

**Results:**

In JSaY program the CS rate increased from 26.6 % (2007–2008) to 40.7 % (2010–2011), against the background rate for CS in Madhya Pradesh, of only 4.9 % (2004–2006). Meanwhile in CY program in Gujarat, the CS rate decreased to 4.3 % (2010–2011) against a background CS rate of 8.1 % (2004–2006).

**Conclusions:**

The findings from India are unique in that they not only point to a significant impact from the introduction of the financial incentives but also how disincentives have an inverse impact on the choice of delivery method.

## Significance

The observations provide the clearest indication of SID influence on the choice of delivery method so far reported from any country.

## Introduction

Medical ethics assume that interventions and procedures are decided purely on the basis of clinical evidence and expected beneficial outcomes for the patient, and certainly not by provider financial considerations. The Federation of Obstetrics and Gynecology (FIGO) Committee for the Ethical Aspects of Human Reproduction stated that: “performing caesarean section for non-medical reasons is not justified” [1]. The Indian Medical Council Act on Profession and Ethics declares that the ‘physician’s prime objective is rendering services to humanity and rewards or financial gains would be subordinate’ [2].

Nevertheless, there exists ample theoretical literature related to the concept of supplier-induced demand (SID) in the health sector. SID is seen as an example of what economists call externalities, an unintended side-effect of an economic interaction, resulting from the lop-sided principal-agent relationship, as it is called in organizational theory, between the patient and the physician. Economic transactions typically involve two parties, a seller and a buyer, whereas in the health sector the role of the buyer (the patient) is mediated by agents (the physician and sometimes the financing agency). The assumption is that the agent acts on behalf of the principal (patient) with only the best interests of the principal as guidance. If the agent is influenced by personal financial incentives to provide more or less services, more expensive services or other services than what would be in the best interests of the principal, it will be a case of SID. Anecdotal evidence exists to support the belief that SID is not uncommon in the health sector and that it may have considerable influence on the quantity and contents of health services. This influence has, however, been difficult to demonstrate with empirical evidence since there are typically many potential confounders which are uncontrollable in observational studies [3].

Leone concluded in an analysis of data from the third District Level Household Survey (DLHS) in India that supply factors may contribute to the rise in the CS rate in India. She noted also that further research is needed to understand whether the demand for institutional deliveries is compromised by pressures for overmedicalization [4].

The increasing obstetric activities, in terms of hospital admissions and surgical interventions, in Denmark were noted by Vallgarda. Her concluding suggestion was that, even if not proven, the increased obstetric activity would seem to be “obstetrician induced labour” [5].

Brazil has one of the highest caesarean section rates in the world, which has stimulated research both related to the health consequences for mother and child, and to the causes behind the high rates. We have reviewed 153 papers on caesarean section in Brazil, of which 17 were found to have a broad relevance to the topic of reasons for choosing caesarean delivery. However, only one of the papers explicitly mentioned the concept of supplier-induced demand and none of the papers presented evidence for impact of provider payment related financial incentives. Health economics literature discusses anecdotal evidence of supplier-induced demand. Data-based empirical evidence is rare, as noted above.

Against the background of the high maternal mortality ratio and the call of the Millennium Development Goal 5 (MDG5), the Central and State governments of India have implemented financial support programs as part of a strategy to increase institutional intrapartum care to reduce maternal mortality. The Indian health care system is characterised by a dominant private health sector largely financed out-of- pocket by users.

## Objectives and Significance

This paper examines two state-led public–private initiatives to raise hospital delivery rates in two neighbouring Indian states—Madhya Pradesh and Gujarat. The federal government program, the JSY, is a conditional cash transfer paid to the mothers when they present to deliver in a facility [6]. The program has been implemented largely through public sector facilities. However, the JSY also allows the accreditation of private hospitals to increase coverage. In Madhya Pradesh (MP), the state initiated a public–private partnership sub-program in 2007. The partnership was called the Janani Sahayogi Yojana (JSaY). Under the JSaY, private sector obstetricians were remunerated by the state for deliveries, notably involving higher reimbursements for caesarean deliveries than for vaginal deliveries, 4530INR and 800 INR respectively, gradually increasing to 5500INR and 1200INR, before being phased out in 2012 [7]. The JSaY program was small in volume; not more than 5000 beneficiaries benefited from the program before it was scaled down [8].

The CY program in neighbouring Gujarat state, which commenced statewide in 2007, is similar to the JSaY program in that it is a state-led public–private initiative, wherein private sector obstetricians are paid by the state to provide delivery services to underprivileged women. An important difference from JSaY, however, is that the reimbursements involve a fixed amount payment for a block of 100 deliveries, regardless of the proportion of vaginal and caesarean deliveries. The payment was calculated on the estimate of an average of 85 normal and 15 complicated deliveries, seven of which would require a caesarean section [9]. The CY program has been larger, with nearly 800,000 beneficiaries to date. The program is still in operation.

Thus, the CY maternal support program has a built-in financial disincentive designed to discourage unnecessary caesarean sections, which are more resource demanding, but not individually compensated for in the package. The JSaY maternal support program, on the contrary, compensates for the extra resources required for an individual caesarean section. Consequently a higher rate of caesarean deliveries will lead to overall higher revenue for the facility. To the extent that the higher reimbursement for a caesarean involves overcompensation, an incentive to supply more exists.

The objective of our study was to compare and assess the impact of the opposite supplier incentives in the two programs. The importance of our study is that it can contribute to a better understanding of what factors are driving the world-wide trend towards increasing caesarean section (CS) rates.

## Methods and Data

Study setting: Gujarat and Madhya Pradesh states in India. Gujarat, a large state on India’s western flank, has a population of 60.1 million inhabitants, over half of whom live in rural areas (57.4 %). The state is socio-economically relatively wealthier than other Indian states. Gujarat ranks as India’s third richest state based on GDP per capita. The state has a strong private health sector, with private obstetricians practicing in small towns in the interior areas of districts. 60 % of all births in the state take place in private health facilities. Madhya Pradesh (MP) is economically poorer, over two-thirds of MP’s 72 million population is rural. A third of all inhabitants live below the poverty line. The private health care sector in Madhya Pradesh is smaller and confined to large cities. A description of each state is provided in Table 1 below.

**Table 1:** State characteristics, Madhya Pradesh and Gujarat

State	Gujarat	Madhya Pradesh
Total population^a^	60.4 million	72 million
Population below poverty line (eligible population)^b^	16.6 %	31.5 %
MMR^d^	122	230
IMR^c^	36/1000	54/1000
Literacy^a^	79 %	70 %

^a^Census of India 2011

^b^Poverty estimates 2011–2012, Govt of India(Tendulkar method) http://planningcommission.nic.in/news/pre_pov2307.pdf

^c^Sample registration system bulletin, Sept 2014, Registrar General, India http://censusindia.gov.in/vital_statistics/SRS_Bulletins/SRS%20Bulletin%20-Sepetember%202014.pdf

^d^Special bulletin on MMR 2010–2012, Registrar General, India

The data from the two programs for this paper was sourced from the Departments of Health and Family Welfare, Government of Gujarat and Government of Madhya Pradesh. Both departments of health collect data from all facilities participating in the respective programs each year. These are shown in Table 2, below. The CS rate was calculated as a proportion of all births that occurred under the respective programs.

**Table 2:** Lower segment Caesarean sections (LSCS) in two programs in Madhya Pradesh (JSaY) and in Gujarat (CY), (in numbers of/rate per hundred)

Year	MP private hospitals	Gujarat private hospitals	MP normal deliveries	Gujarat normal deliveries	MP total LSCS	Gujarat total LSCS	MP rate LSCS	Gujarat rate LSCS
2007–2008	198	865	8080	106,080	2931	7651	26.6	6.7
2008–2009	191	867	14,111	122,217	6711	7846	32.2	6.0
2009–2010	36	721	515	140,132	281	9425	35.3	6.3
2010–2011	44	662	494	150,979	339	6797	40.7	4.3

In each program, privately owned facilities were eligible to participate in the programs if they met certain criteria which included having a qualified obstetrician, a delivery room, an operating theatre and access to an anaesthetist.

Data to estimate background rates of caesarean sections (baseline prior to the programs) in the states were accessed from the District Level Household Survey (DLHS-3) [8]. The DLHS-3 was reported in 2007–2008 but refers to births that occurred between 2004 and 2006 (i.e. the actual survey period), and therefore serves as appropriate baseline for pre-program CS rates.

Descriptive statistical analysis was performed to measure the association between the respective maternal support systems and CS rates by year.

## Results

**Fig. 1:**
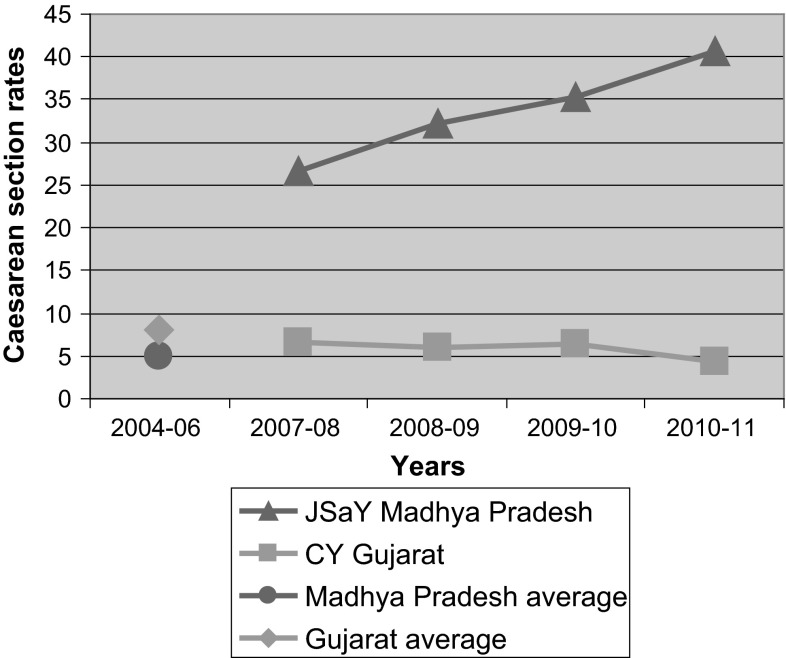
Caesarean section rates in private hospitals in two financial support programs for hospital delivery in two states in India compared with prior state average rates

We report here on how the CS rates among program beneficiaries in private hospitals participating in the two different programs have changed in opposite directions after the programs, with provider financial incentives working in opposite directions, were introduced. As can be seen in the graph (Fig. 1), the trends are dramatically different in the two programs.

In JSaY in MP (program with differential payments for caesarean and vaginal delivery) the CS rate increased over four years, from 26.6 to 40.7 % in the participating private hospitals. This trend is all the more surprising, given that the background rate for caesarean deliveries in Madhya Pradesh, where the JSaY was implemented, was only 4.9 % [10]. Meanwhile in CY program (bundled payment per block of 100 deliveries), the CS rate was close to 6 % with a slightly decreasing trend over the 4 years, with a low 4.3 % rate in 2010–2011. Gujarat state where this program is implemented had a higher than Madhya Pradesh background CS rate of 8.1 % [11].

## Discussion

The two maternal financial support programs differ with respect to their impact on CS rates. The opposite financial incentives in the two programs clearly indicate a remarkable influence of SID on the choice of delivery mode under the two programs in the two states in India. From China a dramatic increase in caesarean deliveries has been reported with possible links to the introduction of the New Rural Cooperative Medical Scheme [11]. Huang et al. [12] concluded that the majority of the extremely high proportion of caesarean deliveries in Anhui province, China, resulted from decisions by the women themselves. Yet, even when the women had the final say, influence from physicians cannot be ruled out. The findings that we report from India are unique in that they not only point to a huge impact from the introduction of the financial incentives but also how disincentives have an inverse impact on the choice of delivery method. The observations provide the clearest indication of SID influence on the choice of delivery method so far reported from any country.

Both programs were for underprivileged women only. Chaturvedi et al. [13] studied written case vignettes for haemorrhage and eclampsia in the JSY program at 73 facilities in Madhya Pradesh and concluded that birth attendants in the facilities had low competence leading to inability to manage obstetric complications, which obviously is a cause for concern in the perspective of increasing CS rates.

The background CS rate in the population was low in both states, 4.9 and 8.1 % in MP and Gujarat respectively (the CS rate in private sector facilities is likely to be higher than the background rates). The CS rates for underprivileged women, before the programs, were lower than for the general population. We estimate that the CS rate for this category of women without access to the programs to be around 2 %. The increase in caesarean deliveries may have saved the lives of poor women, who previously faced financial barriers to access emergency obstetric care. However, we do not believe that the high and increasing CS rate in the Madhya Pradesh JSaY program did reflect a higher and increasing medical need. We suggest that the increase was induced by the pro-CS financial incentives in the JSaY program. Conversely, the CS rate among the eligible beneficiaries in the CY program was much lower, with a tendency to decrease, consistent with the embedded disincentive.

## Conclusions

The findings from our analysis of the two financial support programs to increase institutional intrapartum care to reduce maternal mortality are unique in that they not only point to a significant impact from the introduction of the pro-CS financial incentives, but that they also show how provider payment financial disincentives have an inverse impact on the choice of delivery method. The observations provide the clearest indication of SID influence on the choice of delivery method thus far reported from any country.

Worldwide, CS rates are increasing, a phenomenon coincidental with the drive to increase facility delivery rates in order to meet MDG 5. A growing number of hospitals apply fee systems or provider payment systems as a basis for paying for hospital services, which may or may not introduce financial incentives for supplier-induced demand related to caesarean deliveries. We believe that our observations provide arguments to carefully assess the potential influence of provider payment incentives on the choice of delivery modes when introducing or reforming payment mechanisms.

## References

[1] Schenker JG, Cain JM (1999). FIGO Committee Report: FIGO Committee for the ethical aspects of human reproduction and women’s health. Journal of Gynaecology and Obstetrics.

[2] http://nihfw.nic.in/ndc-nihfw/html/Legislations/TheIndianMedicalCouncilAct.htm.

[3] Morris, S., Devlin, N., Parkin, D., & Spencer, A. (2012). Economic Analysis in Health Care (2nd ed.). Wiley. ISBN 978-1-119-95149-0.

[4] Leone T (2014). Demand and supply factors affecting the rising overmedicalization of birth in India. International Journal of Gynecology and Obstetrics.

[5] Vallgarda S (1989). Increased obstetric activity: a new meaning to “induced labour”?. Journal of Epidemiology and Community Health.

[6] Chaturvedi S, Randive B, Diwan V, De Costa A (2014). Quality of obstetric referral services in India’s JSY cash transfer programme for institutional births: a study from Madhya Pradesh. PLoS One.

[7] http://nihfw.org/pdf/RAHI-I%20Reports/Jabalpur/JABALPUR.pdf.

[8] Sidney, K., de Costa, A., Diwan, V., Mavalankar, D., & Smith, H. (2012). An evaluation of two large scale demand side financing programs for maternal health in India: The MATIND study protocol. *BMC Public Health*, *12*, 699. http://www.biomedcentral.com/1471-2458/12/699. 10.1186/1471-2458-12-699 PMC3488490 22925407

[9] De Costa A, Vora K, Ryan K, Sankara Raman P, Santacatterina M, Mavalankar D (2014). The state-led large scale public private partnership ‘Chiranjeevi Program’ to increase access to institutional delivery among poor women in Gujarat, India: How has it done? What can we learn?. PLoS One.

[10] International Institute for Population Sciences: District Level Household and Facility Survey (DLHS-3), 2007–2008: India. In Mumbai (2010).

[11] Bogg L, Huang K, Long Q, Shen Y, Hemminki E (2010). Dramatic increase of Caesarean deliveries in the midst of health reforms in rural China. Social Science and Medicine.

[12] Huang, K., Tao, F. B., Bogg, L., & Tang, S. L. (2012). Impact of alternative reimbursement strategies in the new cooperative medical scheme on caesarean delivery rates: A mixed-method study in rural China, 2012. *BMC Health Services Research*, *12*, 217. http://www.biomedcentral.com/1472-6963/12/217. 10.1186/1472-6963-12-217 PMC3422992 22828033

[13] Chaturvedi S, Upadhyay S, De Costa A (2014). Competence of birth attendants at providing emergency obstetric care under India’s JSY conditional cash transfer program for institutional delivery: An assessment using case vignettes in Madhya Pradesh. BMC Pregnancy and Child Birth.

